# A telephone survey of cancer awareness among frontline staff: informing training needs

**DOI:** 10.1038/bjc.2011.258

**Published:** 2011-07-12

**Authors:** N Cook, A Hart, K Nuttall, K Simpson, N Turnill, C Grant-Pearce, P Damms, V Allen, K Slade, P Dey

**Affiliations:** 1 School of Postgraduate Medical and Dental Education, University of Central Lancashire, Preston, PR1 2HE, UK; 2 School of Public Health and Clinical Sciences, University of Central Lancashire, Preston, PR1 2HE, UK; 3 Lancashire and South Cumbria Cancer Network, Preston Business Centre, Watling St. Road, Fulwood, Preston, PR2 8DY, UK; 4 Department of Public Knowledge, The Mill, Hexham Business Park, Burn Lane, Hexham, Northumberland, NE46 3RU, UK; 5 HR People Scheme, Lancashire County Council, Staff Training Centre, Moor Street, Kirkham, PR4 2AU, UK; 6 Procurement, Contracting and Market Management, NHS Central Lancashire, Jubilee House, Lancashire Business Park, Centurion Way, Leyland, PR26 6TR, UK; 7 Department of Public Health, NHS North Lancashire, Trust Headquarters, Moor Lane Mill, Moor Lane, Lancaster, LA1 1QD, UK

**Keywords:** neoplasms, health knowledge, attitudes, practice, staff development, risk factors, early detection of cancer

## Abstract

**Background:**

Studies have shown limited awareness about cancer risk factors among hospital-based staff. Less is known about general cancer awareness among community frontline National Health Service and social care staff.

**Methods:**

A cross-sectional computer-assisted telephone survey of 4664 frontline community-based health and social care staff in North West England.

**Results:**

A total of 671 out of 4664 (14.4%) potentially eligible subjects agreed to take part. Over 92% of staff recognised most warning signs, except an unexplained pain (88.8%, *n*=596), cough or hoarseness (86.9%, *n*=583) and a sore that does not heal (77.3%, *n*=519). The bowel cancer-screening programme was recognised by 61.8% (*n*=415) of staff. Most staff agreed that smoking and passive smoking ‘increased the chance of getting cancer.’ Fewer agreed about getting sunburnt more than once as a child (78.0%, *n*=523), being overweight (73.5%, *n*=493), drinking more than one unit of alcohol per day (50.2%, *n*=337) or doing less than 30 min of moderate physical exercise five times a week (41.1%, *n*=276).

**Conclusion:**

Cancer awareness is generally good among frontline staff, but important gaps exist, which might be improved by targeted education and training and through developing clearer messages about cancer risk factors.

Although the United Kingdom has seen marked improvements in cancer survival over the last decade, there is increasing recognition that for survival rates to rival the best in Europe there needs to be a greater emphasis on early presentation (Department of Health, 2011). Cancer mortality is higher in more deprived groups who are more likely to present with late stage disease, (Coleman *et al*, 2001; Macleod *et al*, 2009; National Cancer Intelligence Network, 2010) have lower uptake of screening programmes (McCaffery *et al*, 2002) and are less likely to adopt healthy lifestyle behaviours (Wardle and Steptoe, 2003). Recent surveys have highlighted that the public have low awareness about many cancer warning signs, cancer-screening programmes, and cancer risk factors and that awareness is poorer in lower socio-economic groups (Adlard and Hume, 2003; McCaffery *et al*, 2003; Redeker *et al*, 2009; Robb *et al*, 2009; Waller *et al*, 2009; Keeney *et al*, 2011).

In England, the importance of expedited referral to specialist services by general practitioners in order to reduce treatment delays has been extensively acknowledged through the National Awareness and Early Diagnosis Initiative (Richards, 2009), but the role of other frontline National Health Service (NHS) and social care staff in facilitating access to or signposting the public to primary care services in order to promote earlier consultation has only been more recently recognised. The role of frontline staff as health promoters and role models is more widely acknowledged (Van Leuven, 2006; National Institute for Health and Clinical Excellence, 2011). There is some evidence that staff have limited awareness about the association between lifestyle behaviours and certain cancers, but most of these studies investigating cancer awareness have been undertaken on hospital-based staff or among single professional disciplines (Siriphant *et al*, 2001; Canto *et al*, 2002; Madanat and Merrill, 2002; Tasian *et al*, 2010). Less is known about the awareness of community-based NHS and social care staff about cancer signs and symptoms, cancer-screening programmes and cancer risk factors despite the fact that these staff are often in contact with the most vulnerable and most deprived groups.

Lancashire and South Cumbria Cancer Network undertook a survey of cancer awareness among those involved in provision of healthcare and social care services in two primary care trusts (PCTs) and one local authority. The survey was undertaken to help guide the development of materials and methods for educational training of local frontline community staff and to establish a baseline to assess the effectiveness of future training programmes. The survey used a nationally recognised, validated questionnaire (Robb *et al*, 2009; Waller *et al*, 2009). We report the findings of the survey in this report.

## Materials and methods

Before commencement of the survey, communication briefs were cascaded to staff in the two PCTs, the local authority and independent healthcare employers from which participants were sampled. Work telephone contact details were provided by the organisations for health and social care staff including professional and clinical staff, managers, technicians, administrative and clerical staff, and facilities staff. Staff working in general and dental practices, pharmacies and opticians in the areas covered by the two PCT were also included. Staff job titles were used by the cancer network to categorise staff into one of six staff groups before the survey was conducted. Between 15 March and 16 April 2010, computer-assisted telephone interviews were undertaken by a market research company, Public Knowledge, using the Cancer Research UK Cancer Awareness Measure (CAM).

The CAM includes questions about recall and recognition of cancer signs and symptoms, how soon the participant would go to see a doctor about specific warning signs of cancer, the emotional and practical barriers to accessing primary care services if a cancer warning sign is present, recall and strength of agreement with cancer risk factors, the prevalence of cancer and contribution of different types of risk factors, and awareness about bowel, breast and cervical cancer-screening programmes. Questions are also included on sociodemographic characteristics and personal experience of cancer (self, friends or family). The recall questions about cancer signs and symptoms, and cancer risk factors ask participants to ‘name as many as you can think of’. There are nine questions, which explore participants’ recognition of specified cancer signs and/or symptoms. These questions ask whether, in their opinion, the participant thinks that a specific sign or symptom is or is not a ‘warning sign of cancer’. The participant can answer yes or no. There was no option provided to answer ‘don’t know’ or ‘not sure’, but such a response was recorded by the interviewer when the participant failed to provide a yes or no answer. There are statements about 11 different risk factors. Participants are asked ‘how much do you agree or disagree that each of these can increase the chance of getting cancer according to the following scale?’ and the participant can answer: strongly agree, agree, not sure, disagree or strongly disagree.

To determine the best means of promoting cancer awareness through education and learning, we added a question, which asked staff which were their two most preferred methods for delivery of educational training from a list including e-learning, interactive package with DVD and workbook, facilitated game, posters and booklets, seminar, whole day or half day facilitated training course. A question on smoking status was also added. Participants were asked to confirm their employer and job title. The survey was classed as service evaluation and was deemed not to require NHS ethical approval. Governance approvals and agreement to undertake the survey were obtained from the appropriate organisations. Participants were informed that the findings of the survey may be published. The findings of the survey were disseminated to staff and employing organisations through information on the cancer network website, email cascade and presentations.

### Analysis

Frequency tables were constructed for the overall sample and for subgroups based on staff group, employer and educational attainment. Educational attainment was categorised into three groups based upon the national qualifications framework and framework for higher education qualifications (Qualifications and Curriculum Development Agency, 2006): group 1 included those with honours degrees, master's degrees and doctorates; group 2 included those with A levels, national vocational qualifications, ordinary national certificates and awards given by the Business Technology Education Council, and higher education qualification below degree level. Group 3 included respondents with no formal qualifications or general certificate of secondary education only. Initial analyses comparing staff group as determined by the cancer network, job title and educational attainment suggested a significant number of discrepancies that could not be adequately resolved retrospectively. As educational attainment was collected directly from participants and was not open to interpretation, subgroup analyses were therefore limited to comparisons of awareness across educational attainment groups. The respondent samples for employers other than PCT were too small for meaningful comparisons.

Two of the authors (PD, AH) discussed how relevant the different survey questions were to the objective of the study, which was to help inform training needs. We considered that the opinions of frontline staff on how quickly they would consult a general practitioner and the barriers they perceive they would have accessing primary care may not necessarily reflect the advice that they would give to patients or clients. However, staff could put the public at risk if they failed to give correct advice about warning signs. They could also miss important opportunities to promote healthy lifestyles or preventative strategies if they are unaware of, or failed to acknowledge, cancer risk factors or cancer-screening programmes. These assumptions were discussed with the survey steering group and further analysis focused on responses to questions on cancer warning signs and risk factors and on awareness about screening programmes; responses to the question on preferred training methods were also analysed. Response to other questions can be found in Supplementary tables.

Study data were compared with data from an Office of National Statistics (ONS) Opinions Survey of a representative sample of the United Kingdom population completing the CAM (Wardle *et al*, 2008). This datum is available through the UK Data Archive. An analysis of this latter data set has previously suggested that age, gender, marital status, ethnic group and occupational group are predictors of awareness about cancer warning signs (Robb *et al*, 2009). Our sample has different demographics to the sample from the ONS survey in relation to these predictive factors and therefore weighting was performed. Age group (18–24/25–34/35–44/45–54/55–64/65+), gender (male/female), marital status (married or civil partnership/not married) and ethnic group (white versus black and minority ethnic groups) were used to jointly categorise, where possible, every response in the two data sets; occupational status was not used given the nature of the study. Each response in the ONS data set was then weighted to ensure that the two data sets had similar proportions of each category.

## Results

A total of 5725 contact details were provided to the market research company of which 1061 were either duplicated, incorrect or for ineligible participants giving a potential study population of 4664 contacts. Of 4664 contacts, 359 people could not be contacted, 1330 could not complete the survey during the study period and 2304 refused, mostly because they were too busy. The overall response rate was 671 out of 4664 (14.4%). Of the 671 participants who completed the interview survey during the study period, 85.5% (*n*=574) were female and 86.9% (*n*=583) were aged between 25 and 64 years. Smoking prevalence (13.4%, *n*=90) was lower than the general population (The Health and Social Care Information Centre, 2010), and educational attainment was higher with 37.9% (*n*=254) having a degree or higher degree (Robb *et al*, 2009). The majority of staff (89.6%, *n*=601) were employed by the two PCT. Of the others, 37 (5.5%) were employed by the local authority, 21 (3.1%) by independent health care providers and 12 (1.8%) had other employers (Table 1).

The mean number of warning signs recalled from memory by staff was 3.68 (s.d.=2.05). A swelling or lump was recalled by 482 (71.8%) staff; other signs were recalled by 40% or fewer staff, with cough and hoarseness recalled by only 16.5% (*n*=111). When directly asked their opinion about whether specific signs or symptoms were warning signs of cancer using closed yes/no questions, there was a high level of recognition of most of the nine warning signs of cancer (Table 2). However, a sore that does not heal, cough or hoarseness and unexplained pain were not recognised as being cancer warning signs by 22.7% (*n*=152), 13.1% (*n*=88) and 11.2% (*n*=75) of participants, respectively (Table 2). Staff recognition of cancer warning signs was higher than that reported for a United Kingdom population sample in the ONS Opinions Survey (Robb *et al*, 2009). The weighted analysis comprised 1858 cases comparable to the 596 cases in our data set with all relevant items of demographic information. Staff awareness levels were only slightly attenuated when characteristics of the survey population were taken into account, supporting higher awareness among staff.

The mean number of cancer risk factors recalled from memory by staff was 4.25 (s.d.=2.04). Smoking was most frequently recalled (90.5%, *n*=607). Alcohol was recalled by 62.6% (*n*=420) and all other risk factors by less than 50% of the study sample. When directly asked how much they agreed or disagreed that certain risk factors ‘increased the chance of getting cancer’, the majority agreed or strongly agreed that smoking (98.2%, *n*=659) and passive smoking were risk factors (93.4%, *n*=627). However fewer agreed or strongly agreed that the ‘chance of getting cancer’ was increased by being sunburnt more than once as a child (78.0%, *n*=523), being overweight (73.5%, *n*=493), by drinking more than one unit of alcohol per day (50.2%, *n*=337) or by doing less than 30 min of moderate physical exercise five times a week (41.1%, *n*=276). With the exception of smoking, a higher proportion of participants agreed than strongly agreed with the statements (Table 3). Awareness of the cervical screening and breast screening programmes was high, with over 94% awareness, but relatively poor for bowel cancer-screening with only 415 (61.8%) aware that there was such a programme (Table 2).

Awareness about cancer warning signs and symptoms tended to be slightly higher in those with the highest and lowest educational attainment. Agreement about smoking was similar across all educational attainment groups but agreement about other cancer risk factors tended to be highest in those with highest educational attainment and lowest in those with the lowest educational attainment (Table 4). The most preferred methods for educational delivery to increase cancer awareness were E-learning (42.9%, *n*=288) and a facilitated short game-based intervention (39.2%, *n*=263). Posters and booklets were the least preferred mode of delivery with only 7.7% (*n*=52) of participants selecting this as one of their two preferred methods. The preferred method varied across educational attainment groups. E-learning was preferred most by those with higher educational attainment (58.2%, *n*=134) and DVD and workbook methods were preferred by those with lower educational attainment (46.8%, *n*=52).

## Discussion

To the best of our knowledge, this is the first study to report on cancer awareness in a multidisciplinary group of health and social care staff working within the community using a validated measurement tool. We focused on a limited number of questions included in the CAM because we felt that staff awareness in these areas could directly impact on early prevention and diagnosis among the public. Awareness about cancer warning signs was generally good. Findings suggest higher levels of awareness about warning signs of cancer than in the general public even when demographics are taken into account, although arguably even these high levels of cancer awareness may be insufficient to maximise the benefits to patients, clients and the general public. As in other studies of health professionals (Odusanya and Tayo, 2001; Siriphant *et al*, 2001; Canto *et al*, 2002; Colella *et al*, 2008; Carter *et al*, 2009; Riordain and McCreary, 2009; Ali *et al*, 2010), there are gaps in awareness which raise concern. Although lung cancer is the most commonly occurring cancer, only 86.9% recognised that persistent cough or hoarseness was a cancer warning sign. In comparison, 98.5% recognised a change in a mole as a cancer warning sign despite melanoma being an uncommon cancer. Swelling or lump was recognised by 96.6% of staff and was the most recalled symptom. The least frequently recognised sign was ‘a sore that does not heal’. These variations mirror findings in population samples. High recognition or recall may reflect the success of mass media publicity about breast cancer and malignant melanoma (Robb *et al*, 2009; Waller *et al*, 2009). Poor recognition or recall might reflect the prevalence of these symptoms among the healthy population. For example, cough and hoarseness are a common symptom of self-limiting viral illnesses and it may be less clear when such symptoms should be considered suspicious.

It is important to address confusion and conflicting opinions among staff about lifestyle messages. This would appear to be particularly the case for alcohol consumption; 62.6% recalled alcohol as a risk factor for cancer, which was higher than the proportion who agreed that drinking more than one unit a day was a risk factor on direct prompting (50.2%). The proportion who disagreed that drinking more than one unit of alcohol a day was a risk factor for cancer was similar among those who recalled and those who did not recall it as a risk factor (30.2% *vs* 32.3% df=669, t=1.38, *P*=0.167). Current staff training and public guidelines about alcohol consumption focus on reducing harmful or hazardous drinking and suggest healthy limits in excess of those currently considered to be associated with an increased risk of cancer. Studies have identified staff confusion regarding recommended daily alcohol intake and suggest that staff find it difficult to discuss the risks of a socially acceptable behaviour, of which they themselves may partake (Lock *et al*, 2002; Heather *et al*, 2006). Further work is needed to address these potentially conflicting messages.

Awareness about NHS breast and cervical screening programmes was high in this mainly female cohort. Only two thirds were aware of the bowel screening programme which was introduced in 2006, but which has only recently completed national roll out. In line with other studies, frontline staff had less awareness about cancer risk factors, with the exception of smoking (Odusanya and Tayo, 2001; Siriphant *et al*, 2001; Canto *et al*, 2002; Madanat and Merrill, 2002; Colella *et al*, 2008; Klug *et al*, 2008; Carter *et al*, 2009; Ibrahim and Odusanya, 2009; Riordain and McCreary, 2009; Ali *et al*, 2010; Tasian *et al*, 2010). Studies of cancer awareness among the general public have shown marked socioeconomic variations with poorer awareness of cancer warning signs and cancer symptoms in the most deprived or those with lower educational attainment (Redeker *et al*, 2009; Robb *et al*, 2009; Waller *et al*, 2009; Keeney *et al*, 2011). It has been suggested that this group may have less access to sources of health information and may have fewer opportunities to modify behaviour (Viswanath and Emmons, 2006). However in this study a trend across educational attainment groups was only observed for awareness about cancer risk factors. The lack of a trend of decreasing awareness of warning signs across educational attainment groups may be because health and social care staff are more likely to have received specific training and that they perceive that providing clinical advice is an important part of the role. Those in the lowest and highest educational attainment groups may be in jobs with more patient or client exposure increasing their cancer awareness through the demands and experience inherent in their job role. This study has highlighted that preferred modes of training for staff differ across educational groups. This may reflect access to or skills in using computers. Increasing cancer awareness among frontline staff through education and training is important, but studies of the implementation of brief interventions have highlighted that other factors also need addressing at the same time, including organisational support (Heather *et al*, 2006), provision of time to provide advice and support (Heather *et al*, 2006; Hutchings *et al*, 2006; Groves *et al*, 2010) and skills training in handling emotive subjects (Lock *et al*, 2002; Heather *et al*, 2006; Groves *et al*, 2010).

A major limitation of this study is the very low response rate. This is not unusual for surveys, but it does necessarily limit the generalisability of the results. It is therefore important not to overinterpret the findings, but in the absence of other data about these issues the results can give some important indications of areas for prioritisation of resources and also further research. Given the limited funding available for the study and the timescales for completion, it was anticipated that telephone interviews would facilitate access to a larger number of staff. Telephone interviews are considered an acceptable alternative to face-to-face interviews in public health research and have been used successfully with healthcare staff (Marcus and Crane, 1986; Barriball *et al*, 1996). They are often associated with lower response rates at first contact as observed in this study (O’Cathain *et al*, 2010), which may be due to greater suspicion about, and less engagement with, the survey (Holbrook *et al*, 2003). A further issue identified in this study was that many staff shared the same telephone number, which was also often the contact for patients, meaning that staff could not afford to tie up the phone line in some instances, and also led to refusals due to the large volume of calls received to the same telephone number. It has also been recognised that those participating in telephone surveys are more likely to provide more socially desirable and less extreme responses (Holbrook *et al*, 2003). In this survey, relatively few respondents disagreed with statements, and, with the exception of smoking, few strongly agreed with statements about cancer risk factors (Table 2). The CAM has now been validated for online use, an approach which may increase the response rates among frontline staff.

Despite the low response rate, this survey provides insightful information on staff cancer awareness, which can inform educational and training initiatives; we have already used the study findings locally to inform the development of training resources. High levels of cancer awareness among staff are essential to maximise the impact of public health interventions to promote early consultation and modify lifestyle behaviours. Gaps in awareness in frontline staff are a cause for concern. Further work is particularly needed to develop readily understood messages about many cancer risk factors.

## Figures and Tables

**Table 1:** Demographic characteristics of the study sample (*n*=671)

	***n***	**%**
*Gender*		
Female	574	85.5
		
*Age (years)*		
18–24	66	9.8
25–34	135	20.1
35–44	133	19.8
45–54	214	32.0
55–64	101	15.1
65+	6	0.9
Missing	16	2.4
		
*Marital status*		
Married, civil partnership	387	57.7
Not married	214	10.4
Missing	70	31.9
		
*Ethnicity*		
White	618	92.1
Non-white	43	6.4
Missing	10	1.5
		
*Employer*		
Primary care trust	601	89.6
Local authority	37	5.5
Independent	21	3.1
Other	12	1.8
		
*Educational attainment*		
Group 1	254	37.9
Group 2	288	42.9
Group 3	111	16.5
Missing	18	2.7
		
*Working Status*		
Working full time	420	62.6
Working part time	215	32.0
Other	18	2.7
Missing	18	2.7
		
*Smoking status*		
Smoker	90	13.4
Missing	13	1.9

**Table 2:** Distribution of responses (*n,* %) to statements about cancer warning signs and screening programmes (*n*=671)

	**Yes**	**No/don’t know/not sure**
	***n* (%)**	***n* (%)**
*Number and percentage of the study sample who responded that the following could be warning signs of cancer*		
Unexplained lump or swelling	648 (96.6)	23 (3.4)
Persistent unexplained pain	596 (88.8)	75 (11.2)
Unexplained bleeding	631 (94.0)	40 (6.0)
Persistent cough or hoarseness	583 (86.9)	88 (13.1)
Persistent change in bowel or bladder habits	644 (96.0)	27 (4.0)
Persistent difficulty swallowing	618 (92.1)	53 (7.9)
Change in the appearance of a mole	661 (98.5)	10 (1.5)
A sore that does not heal	519 (77.3)	152 (22.7)
Unexplained weight loss	641 (95.5)	30 (4.5)
		
*Number and percentage of the study sample who responded that the following are existing NHS screening programmes*		
Breast	635 (94.6)	36 (5.4)
Cervical	608 (90.6)	63 (9.4)
Bowel	415 (61.8)	256 (38.2)

**Table 3:** Number and percentage of the study sample who responded that the following could increase the chance of getting cancer (n=671)

	**Strongly agree**	**Agree**	**Not sure**	**Disagree/strongly disagree**
	***n* (%)**	***n* (%)**	***n* (%)**	***n* (%)**
Smoking any cigarettes at all	517 (77.0)	142 (21.2)	7 (1.0)	5 (0.7)
Exposure to other's smoking	287 (42.8)	340 (50.7)	26 (3.9)	18 (2.6)
Drinking >1 unit alcohol per day	52 (7.7)	285 (42.5)	126 (18.8)	208 (30.0)
Eating<5 portions fruit or vegetables per day	38 (5.7)	250 (37.3)	149 (22.2)	234 (34.8)
Eating red/processed meat once per day or more	29 (4.3)	250 (37.3)	162 (24.1)	230 (34.2)
Being overweight (BMI over 25 kg m^−2^)	109 (16.2)	384 (57.2)	71 (10.6)	107 (16.0)
Getting sunburned >once as a child	195 (29.1)	328 (48.9)	59 (8.8)	89 (13.2)
Being >70 years old	63 (9.4)	294 (43.8)	144 (21.5)	170 (25.3)
Having a close relative with cancer	159 (23.7)	392 (58.4)	63 (9.4)	57 (8.4)
Infection with HPV	93 (13.9)	318 (47.7)	212 (31.6)	48 (7.2)
Doing <30 min moderate physical activity 5 times per week	21 (3.1)	255 (38.0)	142 (21.2)	253 (37.7)

Abbreviations: BMI=body mass index; HPV=human papillomavirus.

**Table 4:** Comparison of awareness across educational attainment groups (*n*=653)^a^

	**Group 1 (*n*=254)**	**Group 2 (*n*=288)**	**Group 3 (*n*=111)**
	***n* (%)**	***n* (%)**	***n* (%)**
*Number and percentage of the study sample who agreed that the following could be warning signs of cancer*			
Unexplained lump or swelling	247 (97.2)	275 (95.5)	108 (97.3)
Persistent unexplained pain	239 (94.1)	245 (85.1)	96 (86.5)
Unexplained bleeding	245 (96.5)	260 (90.3)	109 (98.2)
Persistent cough or hoarseness	225 (88.6)	243 (84.4)	99 (89.2)
Persistent change in bowel or bladder habits	249 (98.0)	271 (94.1)	106 (95.5)
Persistent difficulty swallowing	241 (94.9)	254 (88.2)	105 (94.6)
Change in the appearance of a mole	252 (99.2)	281 (97.6)	110 (99.1)
A sore that does not heal	200 (78.7)	219 (76.0)	86 (77.5)
Unexplained weight loss	246 (96.9)	272 (94.4)	105 (94.6)
			
*Number and percentage of the study sample who agreed or strongly agreed that the following could be risk factors for cancer*			
Smoking any cigarettes at all	251 (98.8)	281 (97.6)	109 (98.2)
Exposure to other's smoking	241 (94.9)	269 (93.4)	101 (91.0)
Drinking >1 unit alcohol per day	126 (49.6)	150 (52.1)	53 (47.7)
Eating<5 portions fruit or vegetables per day	136 (53.5)	114 (39.6)	29 (26.1)
Eating red/processed meat once per day or more	124 (48.8)	112 (38.9)	35 (31.5)
Being overweight (BMI over 25 kg m^−2^)	196 (77.2)	214 (74.3)	72 (64.9)
Getting sunburned >once as a child	206 (81.1)	227 (78.8)	77 (69.4)
Being >70 years old	163 (64.2)	138 (47.9)	47 (42.3)
Having a close relative with cancer	214 (84.3)	237 (82.3)	87 (78.4)
HPV infection	179 (70.5)	165 (57.3)	59 (53.2)
Doing <30 mins moderate physical activity 5 times per week	120 (47.2)	114 (39.6)	35 (31.5)
			
*Number and percentage of the study sample who thought that the following NHS screening programmes existed*			
Breast	240 (94.5)	268 (93.1)	109 (98.2)
Cervical	239 (94.1)	258 (89.6)	97 (87.4)
Bowel	154 (60.6)	176 (61.1)	72 (64.9)

Abbreviations: BMI=body mass index; NHS=National Health Service.

a Missing information on educational attainment=18.
