# Supplementary material for: A telephone survey of cancer awareness among frontline staff: informing training needs
Source: Br J Cancer. 2011 Jul 12;105(3):340–5. doi: 10.1038/bjc.2011.258 (PMC3172913; doi:10.1038/bjc.2011.258)
Supplement: Supplementary Tables [file bjc2011258x1.doc]

Supplementary Tables

| Supplementary Table 1: Participant responses to the question ‘If you noticed any of the following unexplained symptoms how soon would you contact your doctor to make an appointment to discuss it, according to the following scales?’ (n=671) | | | | | | | | | | | | |
| --- | --- | --- | --- | --- | --- | --- | --- | --- | --- | --- | --- | --- |
| Time before making an appointment | | | | | | | | | | | | |
| Sign/symptom | <2 weeks | | >=2 weeks, < 6 weeks | | >=6 weeks, < 6 months | | >=6 months to 12 months | | Never | | Don’t Know* | |
|  | N | % | N | % | N | % | N | % | N | % | N | % |
| Unexplained lump/swelling | 546 | 81.4 | 104 | 15.5 | 8 | 1.2 | 4 | .6 | 3 | .4 | 6 | .9 |
| Unexplained pain | 465 | 69.3 | 170 | 25.3 | 15 | 2.2 | 2 | .3 | 8 | 1.2 | 11 | 1.6 |
| Unexplained bleeding | 598 | 89.1 | 59 | 8.8 | 6 | .9 | 0 | 0.0 | 4 | .6 | 4 | .6 |
| Cough or hoarseness | 265 | 39.5 | 326 | 48.6 | 42 | 6.3 | 8 | 1.2 | 21 | 3.1 | 9 | 1.3 |
| Change in bowel/bladder habits | 380 | 56.6 | 232 | 34.6 | 38 | 5.7 | 6 | .9 | 9 | 1.3 | 6 | .9 |
| Difficulty swallowing | 521 | 77.6 | 124 | 18.5 | 8 | 1.2 | 0 | 0.0 | 10 | 1.5 | 8 | 1.2 |
| Change in appearance of a mole | 513 | 76.5 | 125 | 18.6 | 16 | 2.4 | 5 | .7 | 4 | .6 | 8 | 1.2 |
| A sore that did not heal | 406 | 60.5 | 221 | 32.9 | 29 | 4.3 | 2 | .3 | 4 | .6 | 9 | 1.3 |
| Unexplained weight loss | 296 | 44.1 | 260 | 38.7 | 82 | 12.2 | 6 | .9 | 13 | 1.9 | 14 | 2.1 |
| A symptom you thought might be cancer | 595 | 88.7 | 63 | 9.4 | 5 | .7 | 2 | .3 | 1 | .1 | 5 | .7 |

*not read out to participant

| Supplementary Table 2: Participant responses to the question: ‘Could you say if any of these would put you off from going to the doctor?” (n=671) | | | | | |
| --- | --- | --- | --- | --- | --- |
|  | Yes often | Yes sometimes | | No | Don’t know |
|  | n (%) | n (%) | n (%) | | n (%) |
| Too embarrassed | 33 (4.9) | 96 (14.3) | 540 (80.5) | | 2 (.3) |
| Too scared | 39 (5.8) | 148 (22.1) | 482 (71.8) | | 2 (.3) |
| Worried about wasting doctors time | 53 (7.9) | 157 (23.4) | 460 (68.6) | | 1 (.1) |
| Doctor would be difficult to talk to | 22 (3.3) | 74 (11.0) | 568 (84.6) | | 7 (1.0) |
| Difficult to make an appointment | 76 (11.3) | 184 (27.4) | 408 (60.8) | | 3 (.4) |
| Too busy | 47 (7.0) | 191 (28.5) | 433 (64.5) | | 0 (0.0) |
| Too many other things to worry about | 26 (3.9) | 109 (16.2) | 536 (79.9) | | 0 (0.0) |
| Difficult to arrange transport | 5 (.7) | 13 (1.9) | 651 (97.0) | | 2 (.3) |
| Worried what doctor might find | 65 (9.7) | 174 (25.9) | 431 (64.2) | | 1 (.1) |
| Wouldn’t feel confident talking about symptom to a doctor | 15 (2.2) | 51 (7.6) | 603 (89.9) | | 2 (.3) |
